# Incidence and Risk Factors of Postoperative Complications in General Surgery Patients

**DOI:** 10.7759/cureus.30975

**Published:** 2022-11-01

**Authors:** Satish B Dharap, Priya Barbaniya, Shantanu Navgale

**Affiliations:** 1 General Surgery, Topiwala National Medical College & B.Y.L. Nair Charitable Hospital, Mumbai, IND

**Keywords:** postoperative, general surgery, morbidity, predictors, risk factors, complications

## Abstract

Background

Postoperative complications, which are undesirable consequences of surgery, need to be minimized to ensure the quality of surgical care. In this study, we aimed to estimate the incidence and identify the risk factors for postoperative complications which may help in planning appropriate preventive measures.

Methodology

A prospective observational study was conducted in the general surgery department of a tertiary care hospital in a metropolitan city in India. Patients undergoing elective or emergency surgery were included. Patients transferred postoperatively from other hospitals and those undergoing day-care operations or endoscopic procedures were excluded. Age, gender, body mass index (BMI), comorbidities, surgical risk as per American Society of Anesthesiologists (ASA) grading, scheduling of surgery (emergency, semi-emergency, or elective), approach (open or laparoscopic), intraoperative complications, operative blood loss, the extent of surgery (superficial or deep cavity), indication (diagnostic, therapeutic, or palliative), duration of surgery, wound class (clean, clean-contaminated, contaminated, or dirty), and duration of hospital stay in days were recorded. Patients were followed up for 30 days postoperatively for complications (defined as any undesirable, unintended event as a direct result of an operation). Clavien-Dindo classification was used to grade the severity of complications. The chi-square test was used for categorical data, and the t-test was used for numerical data. P-values <0.05 were considered significant.

Results

Postoperative complications were observed in 31.50%; minor complications (Grade I and II) in 19.75% and major complications (Grade III and IV) in 8.0% of patients. Postoperative mortality (Grade V) was 3.75%. Significant risk factors were the presence of comorbidities, higher ASA grade, higher BMI, emergency surgery, open surgery, palliative surgery, deeper cavity surgery, higher intraoperative blood loss, prolonged surgical duration, intraoperative complications, and contaminated surgical wounds. Postoperative complications significantly prolonged the hospital stay.

Conclusions

Understanding risk factors can guide surgeons to adopt appropriate strategic measures to reduce postoperative complications and improve the quality of surgical care. Three key measures emerging from this study are (1) preoperative patient optimization; (2) diligence during surgery to reduce operative time, blood loss, and intraoperative complications; and (3) implementation of infection control practices.

## Introduction

Postoperative complications are undesirable consequences of surgery and are a major area of concern adversely affecting the quality of surgical care and patient safety. These range from seemingly minor incidents which resolve without any harm to more serious incidents which may pose threat to life, need multiple interventions, prolong hospital stay and costs, and may at times cause disability or death [1]. In addition to the physical harm caused to patients, surgical complications can lead to psychological stress and worsen the quality of life [2].

To improve the quality of surgical care, complications need to be minimized. Hence, the incidence and risk factors of postoperative complications need to be understood. However, complications are varied and difficult to capture uniformly. Complications can be specific to the surgical disease, comorbidities, anesthesia, surgical procedure, intensive care interventions, prolonged recumbency, etc. They may affect different body systems such as respiratory, cardiovascular, renal, hepatobiliary, gastrointestinal, neurological, hemopoietic, coagulation, or skin and soft tissues. Complications have been subjectively graded as mild/moderate/severe, minor/major, non-life-threatening/life-threatening, etc. Various scoring systems are used for each complication. Therefore, there is a need to develop a uniform system to capture complications and grade their severity [3].

In 1992, Clavien et al. [3] defined and classified negative outcomes of surgical procedures by differentiating complications, sequelae, and failures: complications as unexpected events not intrinsic to the procedure, sequelae as events intrinsic to the procedure, and failures as events in which the purpose of the procedure is not fulfilled. They proposed a new system of classification that revolutionized the postoperative system of assessment. It graded complications into four grades (grade 4 being death) based on the treatment and interventions needed for the treatment of complications. This grading system was intended to achieve uniformity in reporting complications, compare results in a single center over time, compare results from different centers at the given time, complications of different treatment options, and provide a measuring scale for research [3].

In 2004, the modified version of the above system (the Clavien-Dindo classification) was published [4]. The grading of complications was proposed based on the therapy needed for treating complications. It has seven grades (grades 1 to 5) with two subgroups for grades 3 and 4. Grade 5 denotes death [4]. They reported a five-year experience of using the system which by then had found applications in many fields and had been validated using complex clinical situations from the University of Zurich’s morbidity mortality conferences [5]. Surveys also showed that 90% of surgeons rated the Clavien-Dindo classification to be simple, reproducible, logical, useful, and comprehensive [4]. Hence, the Clavien-Dindo classification system was used in this study.

The objectives of this study were to determine the incidence of postsurgical complications and to study the risk factors associated with postoperative complications using the Clavien-Dindo classification to obtain some clues to plan preventive strategies.

## Materials and methods

The study was conducted in the department of general surgery in a tertiary care medical college hospital and was designed as a prospective observational study after obtaining approval from the institutional ethics committee (Ethics Committee for Academic Research Projects (ECARP); reference number: ECARP/2019/47). It was conducted over a one-year period from March 2020 to February 2021.

The sample size was calculated using the following formula: N = (z)^2^ p (1-p)/e^2^

Where N is the sample size, z at 1.96 (at 95% confidence level according to normal distribution), p at 0.5 (estimated proportion), and e (allowable error) of 0.05. This gave a sample size of 384. Considering the possibility of dropouts, it was increased by approximately 5% to 400.

After obtaining informed consent, patients were enrolled in the study. All patients admitted to the surgical ward for undergoing elective or emergency surgery were included. Patients who were operated on elsewhere and referred were excluded. Daycare and short-stay procedures and gastrointestinal endoscopies were also excluded. Patients’ history, clinical examination, reports of investigations, diagnosis, and operative details were noted. Age, gender, body mass index (BMI, weight in kilograms/square of height in meters), presence of comorbidities, surgical risk as per American Society of Anesthesiologists (ASA) grading, scheduling of surgery (emergency (life-saving surgery), urgent or semi-emergency (early surgery after optimization), and elective (planned)), approach (open or laparoscopic), intraoperative complications if any as documented in surgical notes, operative blood loss in milliliters as estimated and recorded by anesthesiologists, the extent of surgery (superficial limited to the skin and soft tissues of the body wall or deep cavity, mostly abdominal cavity), purpose or indication of surgery (diagnostic, therapeutic, or palliative), duration of surgery and class of surgical wound (clean, clean-contaminated, contaminated, or dirty) were recorded. Patients were followed up for 30 days postoperatively and complications, if any, were noted. The duration of postoperative stay was noted in days.

A complication was defined as any undesirable, unintended event as a direct result of an operation affecting the patient that would not have occurred had the operation gone well as could reasonably be expected. The complication was graded as per the Clavien-Dindo classification (Table 1) [4].

**Table 1:** The Clavien-Dindo classification.

Grade	Description
I	Any deviation from the normal postoperative course without the need for pharmacological treatment or surgical endoscopic and radiological interventions
II	Requiring pharmacological treatment with drugs other than those allowed for grade I complications. Blood transfusion and total parenteral nutrition are also included
III	Requiring surgical, endoscopic, or radiological intervention
III(a)	Intervention not under general anesthesia
III(b)	Intervention under general anesthesia
IV	Life-threatening complications (including central nervous system complications) requiring intensive care unit management
IV(a)	Single organ dysfunction (including dialysis)
IV(b)	Multiorgan dysfunction
V	Death of a patient
Suffix “d”	Complication persistent at discharge (d = disability). Indicative of the need to follow up

The data were tabulated and compiled in Microsoft Excel 2016. SPSS version 16 (IBM Corp., Armonk, NY, USA) was used for analysis. The chi-square test was applied for categorical data while the t-test was used for numerical data. The likelihood ratio test was used for estimating the association between risk factors and complications. A p-value of less than 0.05 was considered significant.

## Results

A total of 400 patients undergoing operations in the department of general surgery were included. Surgeries included surgeries for inguinal hernia, ventral hernia, appendicectomy, colectomy, anterior and abdominoperineal resections, cholecystectomy, excision of lumps, mastectomy, thyroidectomy, splenectomy, distal pancreatectomy, Whipple’s operation, reversal of stoma, feeding gastrostomy or jejunostomy, exploratory laparotomy for intestinal obstruction or peritonitis, and surgeries for skin and soft-tissue infections.

A total of 305 (76.25%) patients were operated on electively; 69 (17.25%) on an emergency basis, and 26 (6.50%) on an urgent (semi-emergency) basis. In total, 55 of 400 (13.75%) were laparoscopic surgeries, 227 (56.75%) were superficial, and 173 (43.2) were deep cavity surgeries. Overall, 379 (94.75%) were therapeutic, 13 (3.25%) were diagnostic, and eight (2%) were palliative procedures.

Age ranged from 13 to 85 years with the mean (±SD) of 42.4 (±15.8) years. The most common age group was less than 40 years (18-40 years) with 201 (50.25%) cases, followed by 41-60 years with 145 (36.25%) cases. There were 211 (52.75%) men and 189 (47.25%) women. The mean weight of study participants was 62.18 ± 9.2 kg (30-78 kg), the mean height was 1.52 ± 0.31 m (1.2-1.74 m), and the mean BMI was 22.38 ± 3.26 kg/m^2^ (17.2-34.81 kg/m^2^). A total of 156 (39%) patients had co-morbidities. The most common comorbidity was hypertension seen in 62 (39.7%) cases, followed by diabetes in 51 (32.6%), chronic kidney disease in 20 (12.8%), bronchial asthma in nine (5.7%), and chronic pulmonary disease in five (3.2%) cases. There were other comorbidities in nine (5.7%) cases, such as hypothyroidism in four cases, HIV infection in three cases, and hepatitis B infection in two cases. A total of 122 (30.50%) patients had undergone some surgery previously. Risk for anesthesia was measured as ASA grade I seen in 244 (61%) cases, followed by grade II in 110 (27.5%), and grade III in 46 (11.5%) cases. The mean duration of surgery was 2.21 ± 1.85 hours (range: 0.5-6 hours). The mean blood loss during surgeries was 176.25 ± 103.17 mL (range: 10-800 mL). Ten cases had intraoperative complications (1.25%) Five cases required inotropic support for hypotension due to septic shock, two had an injury to the bowel during incisional hernia repair, two had raised sugar levels, and one had intraoperative major vessel injury. The class of surgical wounds was clean seen in 231 (57.7%) cases, clean contaminated wounds in 55 (13.7%), contaminated wounds in 53 (13.25%), and infected/dirty wounds in 61 (15.25%) cases. The mean hospital stay was 11.5 ± 8.92 days (range: 1-66 days).

Complications were observed in 126 (31.5%) patients. Table 2 provides the distribution of patients with complications based on the Clavien-Dindo classification. Table 3 shows a bivariate analysis of the association of risk factors with complications. Table 4 depicts the likelihood ratio test.

**Table 2:** Distribution of patients with complications based on the Clavien-Dindo classification.

Clavien-Dindo grade	Frequency N (%)
I	26 (20.6%)
II	53 (42.1%)
IIIA	19 (15.1%)
IIIB	9 (7.1%)
IVA	4 (3.2%)
IVB	0
V	15 (11.9%)
Total	126/400 (31.5%)

**Table 3:** Risk factors for postoperative complications - bivariate analysis.

Variable	Categories	N	Complication present (N = 126)	Complication absent (N = 274)	P-value
Age	<40 years	201	53 (26%)	148	
	41–60 years	145	57 (39%)	88	
	>60 years	54	16 (30%)	38	0.034 <0.05
Gender	Male	211	69	142	
	Female	189	57	132	0.11
Body mass index	Mean ± SD (kg/m^2^)		27.29 ± 1.93	21.16 ± 1.35	0.001
Comorbidities	Present	156	66 (42%)	90	0.01 <0.05
American Society of Anesthesiologists grade	I	244	61 (25%)	183	
	II	110	34 (31%)	76	
	III	46	31 (67%)	15	0.001
Scheduling	Elective	305	73 (24%)	232	
	Urgent (semi-emergency)	26	12 (46%)	14	
	Emergency	69	41 (59%)	28	<0.001
Approach	Laparoscopic	55	11 (20%)	44	
	Open	345	115 (33%)	230	0.048 <0.05
Extent	Superficial	227	52 (23%)	175	
	Deep cavity	173	74 (43%)	99	<0.001
Purpose	Diagnostic	13	1 (8%)	12	
	Therapeutic	379	121 (32%)	258	
	Palliative	8	4 (50%)	4	0.012 <0.05
Duration of surgery (hours)	Mean ± SD		3.24 ± 1.93	1.46 ± 0.83	<0.001
Intraoperative blood loss (mL)	Mean ± SD		229.45 ± 183.42	135.58 ± 74.25	<0.001
Intraoperative complication	Present	10	9 (90%)	1	0.02 <0.05
Class of wound	Clean	231	38 (16%)	193	
	Clean contaminated	55	21 (38%)	34	
	Contaminated	53	25 (47%)	28	
	Infected	61	42 (69%)	19	<0.001
Hospital stay (days)	Mean ± SD		23.14 ± 9.73	9.16 ± 0.83	<0.001

**Table 4:** Risk factors for postoperative complications: likelihood ratio test. *: indicates p-value of <0.05.

Model fitting information	Model fitting criteria			
Model	-2 log likelihood	Chi-Square	df	Significance (p-value)
Intercept only	425.458			
Final	285.143	157.125	345	<0.001
Effect	-2 log likelihood of reduced model	Chi-Square	df	Significance (p-value)
Age	325.068	69.894	67	0.381
Body mass index	311.045	45.241	142	*0.024
Comorbidities	255.227	2.522	1	*0.019
American Society of Anesthesiologists grading (III)	258.835	3.911	1	*0.046
Schedule of surgery (emergency)	255.568	6.941	1	*0.030
Duration of surgery (hours)	356.781	78.254	126	*0.001
Blood loss (mL)	374.325	71.756	132	*0.004
Intraoperative complications	243.217	2.872	1	*0.029
Approach of surgery (open)	258.579	3.405	1	*0.048
Extent of surgery (deep)	259.207	4.032	1	*0.035
Purpose of surgery (palliative)	257.020	14.64	1	*0.004
Class of surgical wound (contaminated-infected)	292.071	36.897	1	*<0.001
Hospital stay	341.315	61.526	124	*0.003

Postoperative complications were significantly associated with various comorbidities and were noted in 41 of 62 (66.13%) hypertensive patients, 42 of 51 (82.35%) diabetic patients, 12 of 20 (60%) patients with chronic kidney disease, three of five (60%) patients with chronic pulmonary disease, and five of nine (55.5%) patients with bronchial asthma. Out of 10 patients who developed intraoperative complications, nine had postoperative complications (90%). Mortality in patients who had intraoperative complications was 50%.

## Discussion

Postoperative complications, although undesirable, are a reality. The incidence of such complications and the risk factors need to be determined for planning preventive strategies. The incidence of postoperative complications after general surgical operations was observed to be 31.50%. As per the Clavien-Dindo classification, the incidence of minor complications (grades I and II) was 19.75% and the incidence of major complications (grades III and IV) was 8.0%. Overall postoperative mortality was 3.75% (grade V). Risk factors associated with postoperative complications were the presence of comorbidities, higher ASA grade, higher BMI, emergency surgery, open surgery, palliative surgery, deeper cavity surgery, higher intraoperative blood loss, prolonged duration of surgery, the occurrence of intraoperative complications, and contaminated and dirty class of surgical wound. In patients with postoperative complications, hospital stay was significantly prolonged.

Contemporary published literature supports our observations. Tevis and Kennedy in a review of literature on postoperative complications in general surgery patients reported an incidence of complications of 5.8-43.5%, with mortality ranging from 0.79% to 5.7%. They reported an overall complication rate of 37%, with 25.7% of complications classified as grade I, 48.6% as grade II, 17.1% as grade III, 5.7% as grade IV, and 2.9% as grade V [1]. A European study in general surgery patients published in 2018 reported an overall complication rate of 12.5%, of which 19% were rated as grade I, 20.7% as grade II, 13.8% as grade IIIA, 27.6% as grade IIIB, 8.6% as grade IVA, and 10.3% as grade V. No grade IVB complications occurred in their investigation [6]. In this study, the overall complication rate was 31.5%, of which grade-wise occurrences were 20.6%, 42%, 15.1%, 7.1%, 3.2%, 0%, and 11.9%, respectively. Both studies did not report grade IVB complications which indicate the death of patients with multiorgan failure (grade V).

A higher incidence of postoperative complications has been reported with advancing age [7]. Unlike the present study, a lower incidence has been observed in women [8]. Women have been observed to report postoperative minor complications such as nausea, vomiting, and headache more often compared to men [9]. The risk of postoperative complications has been reported to be higher in obese individuals [10]. The presence of comorbidities such as cardiac, pulmonary, renal, hepatic, or neurological disease is associated with a significant increase in postoperative complications after major abdominal surgery [11]. In addition, higher postoperative complications have been noted in patients with anemia and perioperative hypoalbuminemia, particularly in low-resource settings [12,13]. A meta-analysis revealed an association between diabetes mellitus with postoperative complications and mortality after non-cardiac surgery [14]. Perioperative hyperglycemia is associated with increased rates of surgical-site infection (SSI), myocardial infarction, stroke, and death [15]. Since 1996, the ASA perioperative risk grading has been a predictor of postoperative complications and outcomes [16]. This study also found higher complications among patients with comorbidities and higher ASA grades.

The study results noted a higher incidence of complications after emergency surgery. Emergency surgery has been observed to have a higher incidence of SSI, pneumonia, myocardial infarction, major bleeding, and stroke with higher subsequent mortality [17]. Focused attention to preoperative optimization may be needed for quality improvement [17]. In this study, the minimal access or laparoscopic approach had fewer complications in comparison to open surgery. This could be because of selection bias as laparoscopic surgeries are usually performed in patients who have good cardiorespiratory reserves and with localized and early disease. However, several papers on different surgeries have also stated that minimal access surgery has fewer postoperative complications [1]. Abdominal surgeries involving the peritoneal cavity (deep cavity surgeries) had a significantly higher incidence of postoperative complications. Gastrointestinal surgeries have been reported to have a significantly higher incidence of SSI, particularly deep or organ space infection [18]. Surgeries in infected or dirty sites or cavities have been observed to have the highest incidence of complications (48%) compared to contaminated wounds (29%) or clean wounds (17%) [18]. Palliative surgeries, done usually for advanced disease (cancer) or in patients unfit due to comorbidities, were associated with significantly higher postoperative complications. This has been reported by other studies as well [19,20].

Longer duration of surgery and increased blood loss during surgery enhance the negative impact on postoperative complications. While studying adverse events in hospitalized patients, it has been observed that postoperative complications in longer surgeries were due to increased blood loss, longer anesthesia requirements, and the indication of the surgery itself [21]. Although the Clavien-Dindo classification does not capture intraoperative complications, in this prospective study, the occurrence of any complication during surgery was specifically noted. Duration of surgery, amount of blood loss, intraoperative hypotension, and transfusion requirement can serve as surrogate markers for the complexity of the surgery and intraoperative complications. In a systematic review and meta-analysis published in 2018, prolonged operative duration was found to be associated with complications [22]. Intraoperative hypotension and blood transfusions have also been observed to be associated with morbidity and mortality [23,24]. Therefore, all efforts must be made to control bleeding during surgery. A multimodal and multidisciplinary approach has been recommended. Correction of anemia and optimization of erythropoiesis, management of antiplatelet and anticoagulant therapy, and risk stratification should be done in the preoperative phase. Use of antifibrinolytics (tranexamic acid), use of a tourniquet and meticulous surgical technique, use of electrosurgery and topical agents for hemostasis, use of cell savers, and autologous blood transfusion can be practiced by surgeons. Anesthesiologists can help in controlling blood loss by implementing permissive hypotension and hemostatic resuscitation, appropriate patient positioning, preventing hypothermia, and maintaining ventilation. The availability of viscoelastic assays in patients undergoing complex surgeries is also a useful strategy [25]. A significant increase in hospital stay has been reported in patients undergoing more complex surgeries and with higher Clavien-Dindo grades similar to the observations of this study [6].

The Clavien-Dindo classification was initially reported for general surgical patients. It has been used in many other surgical settings and is useful. In one paper, while applying it to emergency surgical patients, complications were reported in nearly 26% of patients, however, the authors recommended that preoperative organ dysfunction should be taken into account when defining postoperative grade IV complications [26].

The study has some limitations. The patient population was rather heterogenous and included all patients operated in a general surgical department including operations for different diseases, on different organs, and performed by different surgeons with different levels of experience. Such a design was necessary to identify different risk factors. The incidence will be different if a population is more homogeneous. The follow-up period was limited to 30 days postoperatively. Delayed complications and long-term effects of complications on quality of life have not been studied. Complications such as recurrence of hernia, chronic pain, development of incisional hernia, recurrence, and survival in malignant cases take much longer to develop and need long-term follow-up. However, most serious complications do occur in the early phase within 30 days [27]. Complications can also result from medical errors, which have not been separately recorded and studied in this study [28,29]. Although the Clavien-Dindo classification is simple, reproducible, logical, useful, and comprehensive and has been validated and found applicable worldwide in many fields of surgery, the incidence of a specific complication cannot be estimated using the classification, for example, the incidence of deep vein thrombosis or wound dehiscence. Such events need to be recorded separately.

## Conclusions

Postoperative complications that contribute to negative surgical outcomes were observed in over 30% of general surgery patients. The presence of comorbidities, higher ASA grade, higher BMI, emergency surgery, higher intraoperative blood loss, prolonged surgical duration, intraoperative complications, and contaminated surgical wounds were factors associated with postoperative complications that could potentially be modified to reduce the risk. The negative impact of comorbidities can be reduced by thorough preoperative assessment and treatment. Perioperative management of comorbidities requires good communication and coordination among physicians, anesthesiologists, and surgeons. Preoperative weight reduction should be considered in obese patients whenever feasible. Emergency surgery needs to be conducted expeditiously for immediate life-threatening problems such as hemorrhagic shock. In others, correcting metabolic derangements and optimizing body physiology takes precedence. The operation itself, particularly when complex, needs to be planned and executed meticulously with expert assistance to reduce the operative time, intraoperative blood loss, and intraoperative complications. Appropriate infection control strategies should be considered while managing contaminated surgical sites.
